# HAG-NET: Hiding Data and Adversarial Attacking with Generative Adversarial Network

**DOI:** 10.3390/e26030269

**Published:** 2024-03-19

**Authors:** Haiju Fan, Jinsong Wang

**Affiliations:** College of Computer and Information Engineering, Henan Normal University, Xinxiang 453007, China; wangjinsong@stu.htu.edu.cn

**Keywords:** adversarial attack, watermarking, generative adversarial networks, deep learning, image information entropy

## Abstract

Recent studies on watermarking techniques based on image carriers have demonstrated new approaches that combine adversarial perturbations against steganalysis with embedding distortions. However, while these methods successfully counter convolutional neural network-based steganalysis, they do not adequately protect the data of the carrier itself. Recognizing the high sensitivity of Deep Neural Networks (DNNs) to small perturbations, we propose HAG-NET, a method based on image carriers, which is jointly trained by the encoder, decoder, and attacker. In this paper, the encoder generates Adversarial Steganographic Examples (ASEs) that are adversarial to the target classification network, thereby providing protection for the carrier data. Additionally, the decoder can recover secret data from ASEs. The experimental results demonstrate that ASEs produced by HAG-NET achieve an average success rate of over 99% on both the MNIST and CIFAR-10 datasets. ASEs generated with the attacker exhibit greater robustness in terms of attack ability, with an average increase of about 3.32%. Furthermore, our method, when compared with other generative stego examples under similar perturbation strength, contains significantly more information according to image information entropy measurements.

## 1. Introduction

Conventional image-based watermarking techniques typically alter the pixel values or structure of an image, potentially making the watermark detectable or modifiable by attackers. In contrast, recent research introduces a method that incorporates adversarial perturbations into watermark samples, enabling the watermark to disrupt the classification process of the target neural network. This not only enhances the robustness and stealthiness of the watermark but also safeguards the integrity and security of the image data. Consequently, even if attackers attempt to manipulate the image to compromise the watermark, it can effectively maintain the integrity and security of the image data, thus paving the way for new possibilities in covert communication and watermarking. As a result, certain watermarking techniques, such as ADV-EMB [1] and JAS [2], have been developed to integrate adversarial perturbations, which are adversarial to steganalysis networks, with embedding distortions.

Unfortunately, the aforementioned novel watermarking techniques primarily focus on making the watermark resistant to detection by target steganalysis while neglecting the need to protect the information of the carrier image itself. With the rapid development of big data applications, a multitude of security risks for users have emerged. Image data, being a pivotal component of big data, frequently harbor personal information such as portraits, addresses, income, and interests. When identified by target classification networks utilized in big data applications, it not only exposes the risk of personal information leakage (including spam messages and telecom fraud) but also potentially jeopardizes user safety. Hence, it becomes imperative to integrate watermarking with adversarial attack techniques.

**Figure 1:**
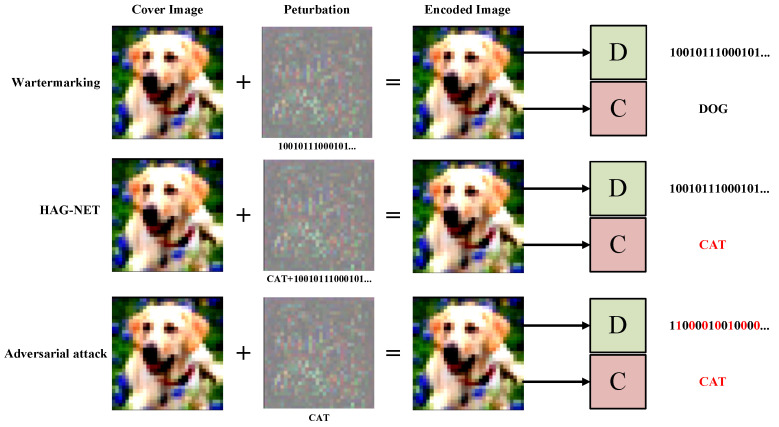
Schematic diagram illustrating the variance in perturbations generated by various generative methods. The string represented beneath these perturbations denotes the secret data or the targeted class of the adversarial attack embedded within. *D* represents the decoder, responsible for decoding the secret data, while its output represents the decoded secret information. *C* denotes the target classification network, with its output indicating the classified prediction, and the red section highlights inaccuracies in the prediction.

Therefore, we propose a novel dynamic data hiding method called HAG-NET (Hiding data and Adversarial attacking with Generative adversarial Network), which is capable of directly generating Adversarial Steganographic Examples (ASEs). In contrast to previous approaches, the ASEs generated by HAG-NET can adversarially attack the target recognition network while concealing the secret information, as depicted in Figure 1. HAG-NET employs three convolutional networks to generate ASEs. The encoder network takes the cover image and secret message (random binary bit string) as input and outputs the ASEs. Conversely, the decoder network accepts the ASEs and attempts to recover the embedded secret message.

We summarize HAG-NET’s contributions as follows:

- In contrast to prior research, we propose a novel Generative Adversarial Network (GAN) framework named HAG-NET, wherein a generator, discriminator, and an attacker are trained jointly. Through co-training with the attacker, HAG-NET further enhances the robustness of the watermark.
- Building upon secret message embedding and resistance to steganalysis, HAG-NET achieves protection against detection for the carrier data.
- The information contained in the adversarial embedded disturbance generated by our method is superior to those of others with the same intensity.

## 2. Related Work

### 2.1. Adversarial Examples

Adversarial Examples (AEs) possess the capability to significantly reduce the recognition accuracy of target classification networks by introducing minimal perturbations to the original image. This phenomenon was first identified by Christian et al. in [3,4]. Subsequently, various adversarial attack algorithms have been proposed, broadly categorized into two groups: white-box attacks, where all data of the target model are known, and black-box attacks, where the training process and parameters of the target model are unknown. Among these, white-box attacks are most relevant to our proposed method.

Ian J Goodfellow et al. [4] introduced FGSM, which calculates gradients through backpropagation to effectively generate perturbations. Madry et al. [5] proposed PGD based on FGSM, which updates the perturbations with a smaller step size in iterations, resulting in steady loss reduction during the iteration process and yielding improved attack effectiveness and visual quality. The Adversarial Examples generated by PGD demonstrate outstanding attack capabilities across various undefended and defended target classification networks.

In [6], an algorithm based on Zero-Order Optimization (ZOO) is proposed to approximate the gradient of the target model. Additionally, in [7], an optimization-based C&W attack algorithm is introduced with the aim of optimizing the objective equation ${\left\|\delta \right\|}_{p}+c\cdot f(x+\delta )$, where f(·) is the objective function measuring the attack effect, δ represents the generated disturbance, c is a constant greater than 0 and p∈(0,2,∞). The disturbance δ for optimal attack and visual quality is determined by optimizing the target equation under the constraint of ${\left\|\cdot \right\|}_{p}$ and *c*.

### 2.2. Watermarking

The most relevant methods are as follows: (1) Least-Significant Bit (LSB) [8]: Information hiding involves systematically modifying the least significant bits of selected pixels in the cover image based on the secret information. Several watermarking methods based on LSB are introduced in [9,10]. Although these methods ensure that perturbations caused by pixel modifications are imperceptible to the naked eye and result in excellent visual quality of stego examples, the systematic alteration of pixel values affects the image’s statistics, making such stego examples easily identifiable [11]. (2) Other watermarking algorithms differ in their approach to measuring minimum distortion during encoding. For example, Highly Undetectable steganography (HUGO) [8] measures the distortion degree of the encoded image by calculating the weights of local neighboring pixels of the cover image. Wavelets Obtained Weight (WOW) [12] employs a bank of directional filters to penalize distortion in predictable regions of the image. S-UNIWARD [13] resembles WOW but can be utilized to embed in arbitrary domains.

### 2.3. Generative Approach

Generative networks have gained considerable popularity for data generation and have witnessed significant advancements in recent years. Saeed et al. [14] introduced the concept of generative adversarial perturbations utilizing U-Net [15] and ResNet [16], laying the foundation for subsequent research in this domain. Expanding on their work, Mao et al. [17] further improved the realism of generated Adversarial Examples (AEs), enhancing their visual perception. Additionally, ADV-GAN in [18], achieved successful targeted attacks on black-box models by incorporating distillation networks and dynamic queries. AI-GAN [19] achieves adaptive attacks against arbitrary target classes.

Generative networks have also found application in watermarking, with prior works typically integrating Deep Neural Networks (DNNs) as a specific component within the overall process. In [20], DNNs were solely used to quantify the watermark strength of each image region, while [21,22] employed DNNs either as encoders or decoders. Reference [23] introduced HiDDeN, the first to fully model the steganographic system using DNNs. Fan, Zexin et al. [2] proposed Joint Adversarial Steganography (JAS), combining adversarial steganography with handcrafted adjustment strategies to design a more secure embedding distortion technique. Tang, Weixuan et al. [1] introduced ADV-EMB, which closely resembles our approach. It adjusts modifications of carrier image units based on gradients propagated from the target steganalysis, deceiving the steganalysis while embedding covert information. However, none of the aforementioned methods provide protection for the carrier data while ensuring watermark security. Moreover, alternative forms of watermarking methods have been explored. In [24], a generative network was trained to conceal an entire image within another image. Uchida et al. [25] embedded watermarks into the trained neural network weights, whereas Fang et al. [26] embedded messages into text carriers using an LSTM network.

**Figure 2:**
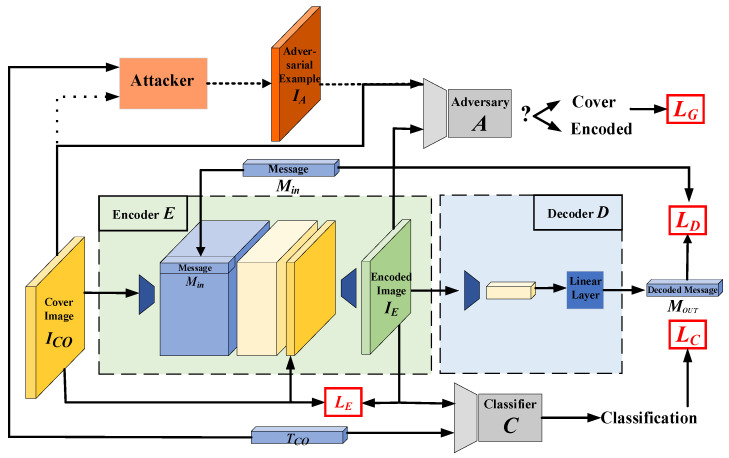
The framework of HAG-NET: the encoder *E* receives the cover image *I_CO_* and the secret message *M_IN_* to generate encoded image *I_E_*; the decoder *D* recovers *M_IN_* from *I_E_* and outputs the decoded message *M_OUT_*; the attacker generates adversarial example *I_A_*. The adversarial discriminator *A* receives *I_CO_* or *I_A_* and *I_E_* to predict whether the input has been encoded; the target classifier *C* predicts the classification of *I_E_*. The loss function *L_E_* is the pixel-level difference between *I_E_* and *I_CO_*; the loss function *L_C_* is used to optimize the ability to resist attacks. The loss function *L_G_* provides adversarial loss for *E*. The loss function *L_D_* minimizes the difference between *M_IN_* and *M_OUT_*. The dashed line indicates that data are transferred according to the settings.

HAG-NET adopts an end-to-end training approach, akin to the HiDDeN framework proposed in [23] and based on GAN. In contrast to previous studies, HAG-NET operates on a query attack and generative watermarking basis, facilitating both data hiding and adversarial attack functionalities by adaptively generating ASE. This capability enables the creation of adversarial steganography while simultaneously ensuring protection for the carrier data. Adversarial Steganographic Examples generated by HAG-NET exhibit excellent attack efficacy against both target and non-target adversaries. The framework of HAG-NET is illustrated in Figure 2.

## 3. Our Approach

### 3.1. Problem Description

Consider a classification network, denoted as C, trained on dataset $X\in \;R^{n}$, where *n* represents the dimension of inputs. During non-target attacks, ${(x}_{i},y_{i})$ denotes the $i^{th}$ instance in the training data, where $x_{i}\in X$ is a cover image generated from an unknown distribution, and $y_{i}$ represents the correct classification of $x_{i}$. Conversely, during target attacks, ${(x}_{i},t_{i})$ denotes the $i^{th}$ instance in the training data, where $t_{i}$ represents the target classification to be attacked for $x_{i}$, and $t_{i}\neq y_{i}$. $M_{IN}\in {\left[0,1\right]}^{L}$ be a binary secret message of length *L*. An instance and a secret message ($M_{IN}$) are used to generate an Adversarial Steganographic Example (ASE), denoted as $E({(x}_{i},y_{i}),M_{IN})=I_{E}$ or $({(x}_{i},t_{i}),M_{IN})=I_{E}$, which resembles $x_{i}$ based on a certain distance measure and is adversarial to *C*. The decoder *D* attempts to recover $M_{IN}$ from the received Adversarial Steganographic Example ($I_{E})$ as the decoded message ($M_{OUT}$). We aim for classification *C* to produce incorrect predictions when the Bit Error Rate between $M_{OUT}$ and $M_{IN}$ is below a certain threshold.

1. When the input is ${(x}_{i},y_{i})$, making $C(E({(x}_{i},y_{i}),M_{IN}))\neq y_{i}$, namely $C\left(I_{E}\right)\neq y_{i}$, it is referred to as the non-target attack;
2. When the input is ${(x}_{i},t_{i})$, making $C(E({(x}_{i},t_{i}),M_{IN}))=t_{i}$, namely $C(I_{E})=t_{i}$, it is referred to as the target attack.

### 3.2. Loss Functions

As illustrated in Figure 1, the HAG-NET network consists entirely of Deep Neural Networks (DNNs). Its main components include the following: encoder $E_{\theta }$, decoder $D_{\emptyset }$, adversarial discriminator $A_{\gamma }$, attacker, and target classifier $C_{\beta }$, where θ, ∅, and γ represent the trainable parameters in $E_{\theta }$, $D_{\emptyset }$, and $A_{\gamma }$ respectively, and β denotes the pre-trained parameters of $C_{\beta }$. To facilitate the mathematical description of the loss function, in the remainder of this article, we use the symbol $I_{CO}$ instead of $x_{i}$ and the symbol $T_{CO}$ instead of $y_{i}$, $t_{i}$, namely $T_{CO}=y_{i}$ or $T_{CO}=t_{i}$.

The Encoder $E_{\theta }$ receives $I_{CO}$, $T_{CO}$ and $M_{IN}\in {\left[0,1\right]}^{L}$ to generate $I_{E}$. The Adversarial Steganographic Example $\left(I_{E}\right)$ generated by $E_{\theta }$ aims to resemble $I_{CO}$ or $I_{A}$ as closely as possible. We employ the $L_{2}$ norm distance to quantify the difference between $\tilde{I}\in \left\{I_{CO},I_{E}\right\}$ (when attackers are not involved in the training process) or $\tilde{I}\in \left\{I_{A},I_{E}\right\}$ (when attackers are involved in the training process), denoted as $L_{E}\left(\cdot \right)$, namely:

(1) $$L_{E}\left(\tilde{I}\right)=\frac{{\left\|I_{CO}-I_{E}\right\|}_{2}^{2}}{\left(c\times H\times W\right)}\;or\;L_{E}\left(\tilde{I}\right)=\frac{{\left\|I_{A}-I_{E}\right\|}_{2}^{2}}{\left(c\times H\times W\right)}$$

The variables W and H represent the pixel width and pixel height of the cover image respectively, while *c* denotes the number of channels in the cover image. Therefore, c×H×W denotes the total number of pixels in the carrier image.

The Adversarial Discriminator $A_{\gamma }$ takes as input the sample $\tilde{I}\in \left\{I_{CO},I_{E}\right\}$ or $\tilde{I}\in \left\{I_{A},I_{E}\right\}$ and predicts whether the input image is encoded. The prediction result of $A_{\gamma }$, denoted as $A\left(\cdot \right)\in [0,1]$, indicates the confidence with which $A_{\gamma }$ considers the input sample to be $I_{E}$. Here, we employ Binary Cross-Entropy (BCE) to measure this classification loss $L_{A}$, expressed as follows:

(2) $$\begin{array}{c} L_{A}\left({A(I}_{co}),A(I_{E})\right)=log\left(1-{\left(1+e^{-A\left(I_{co}\right)}\right)}^{-1}\right)+log\left({\left(1+e^{-A\left(I_{E}\right)}\right)}^{-1}\right)\;\\ or\;\\ L_{A}\left({A(I}_{A}),A(I_{E})\right)=log\left(1-{\left(1+e^{-A\left(I_{A}\right)}\right)}^{-1}\right)+log\left({\left(1+e^{-A\left(I_{E}\right)}\right)}^{-1}\right) \end{array}$$

And the adversarial loss $L_{G}$ from $A_{\gamma }$ is

(3) $$L_{G}\left(A\left(I_{E}\right)\right)=log\left(1-{\left(1+e^{-A\left(I_{E}\right)}\right)}^{-1}\right)$$

The Decoder $D_{\emptyset }$ receives $I_{E}$ and then outputs $M_{OUT}\in {\left[0,1\right]}^{L}$, which is decoded from $I_{E}$. It is important to note that the lengths of $M_{IN}$ and $M_{OUT}$ should be the same. Similar to $L_{E}$, we also employ the $L_{2}$ norm distance to measure the bit-level difference between $M_{IN}$ and $M_{OUT}$. The loss function $L_{D}$ is defined as follows:

(4) $$L_{D}\left(M_{in},M_{out}\right)={\left\|M_{in}-M_{out}\right\|}_{2}^{2}/L$$

Target Classification Network $C_{\beta }$ will classify $I_{E}$ and obtain the classification prediction $P_{i}\in {\left[0,1\right]}^{10}$, where $P_{i}$ represents the classification prediction of the ith example in the current batch. Depending on the attack mode (target attack or non-target attack), $C_{\beta }$ will receive different $T_{CO}$ values ($T_{CO}=y_{i}$ or $T_{CO}=t_{i}$) to calculate $L_{C}$ and the loss function $L_{C}$ is divided into two situations as follows:

1. Non-target attack: In this scenario, the content of $T_{CO}$ is $y_{i}$, which represents the correct classification of $I_{CO}$. Let $P_{real}$ be equal to the ${y_{i}}^{th}$ dimensional vector in $P_{i}$, and let $P_{other}$ be the vector in vectors in $P_{i}$ excluding the ${y_{i}}^{th}$ dimensional vector. Therefore, $P_{real}$ represents the confidence level of that $C_{\beta }$ in considering $I_{E}$ to belong to the ${y_{i}}^{th}$ class. Conversely, $P_{other}$ represents the confidence level of all other classes in $P_{i}$ except the ${y_{i}}^{th}$ class, Thus, we have
  (5) $$L_{C}\left(I_{E},T_{co}\right)=max\left(P_{real}-max\left(P_{other}\right),0\right)$$
2. Target attack: In this scenario, the content of $T_{CO}$ is $t_{i}$, which represents the target classification of attack. Let $P_{target}$ be equal to the ${t_{i}}^{th}$ dimensional vector in $P_{i}$, and let $P_{other}$ be the vector in $P_{i}$ excluding the ${t_{i}}^{th}$ dimensional vector. Therefore, $P_{target}$ represents the confidence level of that $C_{\beta }$ in considering $I_{E}$ to belong to the ${t_{i}}^{th}$ class. Conversely, $P_{other}$ represents the confidence level of all other classes in $P_{i}$ except the ${t_{i}}^{th}$ class, Thus, we have
  (6) $$L_{C}\left(I_{E},T_{CO}\right)=max\left(max\left(P_{other}\right)-P_{target},0\right)$$

Our objective is to maximize the effectiveness of the attack during embedding. To achieve this, we employ Stochastic Gradient Descent (SGD) to minimize the objective function by optimizing θ and ∅ based on the distribution of $I_{CO},{\;M}_{IN}$, and $T_{CO}$, namely

(7) $$\begin{array}{c} minimize\;E_{I_{CO},{M_{in},T}_{co}}\left[L_{D}\left(M_{IN},M_{OUT}\right)+\lambda_{E}L_{E}\left(\tilde{I}\right)+\lambda_{G}L_{G}\left(I_{E}\right)+\lambda_{C}L_{C}\left(I_{E},T_{CO}\right)\right]\\ subject\;to\;L_{D}\left(M_{IN},M_{OUT}\right)\leq d \end{array}$$

where $\lambda_{E}$, $\lambda_{G}$ and $\lambda_{C}\in \left[0,1\right]$ are hyperparameters controlling the weights of different losses. The constant d is a small number, set to $10^{-3}$. Following the GAN concept, we concurrently train the parameter γ. The adversarial discriminator $A_{\gamma }$ aims to minimize the $L_{A}$ loss on the same distribution:

(8) $$\begin{array}{c} minimize\;E_{I_{co},{M_{in},T}_{co}}\left[L_{A}\left(A\left(\tilde{I}\right)\right)\right]\\ subject\;to\;L_{D}\left(M_{IN},M_{OUT}\right)\leq d \end{array}$$

### 3.3. Architecture of HAG-NET

**Figure 3:**
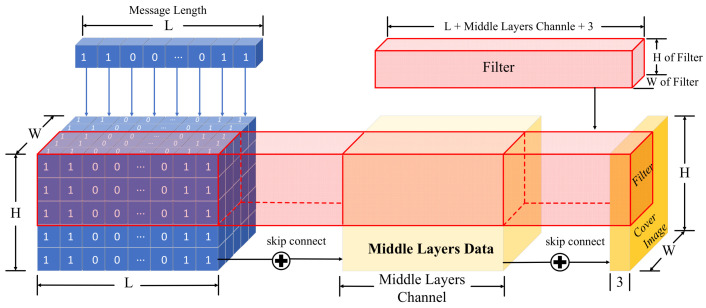
Schematic diagram of the skip connection of the secret message in middle layers, where secret message is *M_IN_*, cover image is *I_CO_*, and the expanded secret message will be the same size as *I_CO_* and the middle layers data.

The Encoder $E_{\theta }$: Initially, $E_{\theta }$ use a convolutional layer to downsample the data from $I_{CO}$ and subsequently generates intermediate layer data. Before passing the data to the next layer, the secret message $M_{IN}$ is expanded to match the size of the intermediate layer data and is connected to the data based on channel dimensions. This process ensures that each convolutional layer’s filter in the subsequent stages has complete access to $M_{IN}$, enabling the encoder $I_{CO}$ to embed $M_{IN}$ into any spatial position of $I_{CO}$. Following the upsampling operation of the subsequent convolutional layers, the data are transformed to match the size of $I_{CO}$. To ensure that $I_{E}$ closely resembles $I_{CO}$ and is distinguishable from an autoencoder primarily focused on dimensionality reduction and $I_{CO}$ reconstruction, we bypass and link $I_{CO}$ with the data prior to the output layer. The schematic diagram illustrating this process is depicted in Figure 3.

The Decoder $D_{\emptyset }$ and The Adversarial Discriminator $A_{\gamma }$: In contrast to the encoder $E_{\theta }$, the channels of intermediate data generated by $D_{\emptyset }$ and $A_{\gamma }$ have the same length *L*. Following global spatial pooling and prediction with fully connected linear prediction layers, the output $M_{OUT}$ of $D_{\emptyset }$ matches the size of the secret message $M_{IN}$. The output of $A_{\gamma }$ is a value indicating the likelihood that $A_{\gamma }$ considers the input sample to be $I_{E}$.

Target Classification Network *C:* We pre-trained some classification networks on CIFAR-10 and MINST datasets, including Model A [22], Model B [6], ResNet32 [22], Wide ResNet34 (WRN34), and the All-Convolution Network [24]. To achieve higher classification accuracy, we made some modifications to the ResNet32 and WRN34, based on their original network architectures. The network architectures are presented in Table 1 below.

Attacker: We input $I_{CO}$ and $T_{CO}$ into the attacker based on PGD to generate $I_{A}$ through targeted or non-targeted attacking. The iterative principle is shown as follows:

(9) $$I_{A}^{N+1}=\prod_{I_{co}+S}^{I_{A}^{0}=I_{co}}\left\{I_{A}^{N}+\alpha sign\left[\nabla_{I_{A}^{N}}J\left(\beta ,I_{A}^{N},T_{co}\right)\right]\right\}$$

where *J* (∙) is the cross-entropy loss function, *β* is the parameter of the pre-trained target classifier, *α* is the amplitude of image pixel update in each iteration, *S* is the maximum perturbations strength, α is the iterative maximum perturbation intensity, and $\nabla_{I_{A}^{N}}$ represents the gradient of $I_{A}^{N}$; these gradients inform us of the direction in which $I_{A}^{N}$ should move to decrease the loss function. Each iteration updates $I_{A}^{N}$ to $I_{A}^{N+1}$, and the final output is the adversarial sample $I_{A}$, which participates in the training process of HAG-NET. Both images $I_{A}$ and $I_{CO}$ have the same size, and the adversarial image $I_{A}$ is then forwarded to $A_{\gamma }$ for classification using an alternative approach. The pseudo-code flow is outlined in Algorithm 1.
**Algorithm 1** uses PGD attack method to generate AE**Input:**Cover image $I_{CO}$, the parameters β of pre-trained target classification network, the maximum iterations *T*, the perturbation step size α, and the maximum perturbation range *S***Output:**Adversarial example *I_A_*1:$I_{A}^{0}\leftarrow I_{CO}$//The initial adversarial example is the cover image.2:*for*    i=0:T *do*3:      $g^{i}=\nabla_{I_{CO}}J\left(\beta ,I_{A}^{i},T_{CO}\right)$//Get the gradient at the current iteration exampl.4:      $d^{i}=\alpha \times sign(g^{i})$//Get the perturbation magnitude at the current iteration.5:    *if* $I_{A}^{i}+d^{i}\in I_{CO}+S$ *then*6:            $I_{A}^{i+1}=I_{A}^{i}+d^{i}$//Update the perturbed image7:    
*Else*
8:            $I_{A}^{i+1}=Clip(I_{A}^{i}+d^{i},I_{CO}+S)$//Confine the perturbations to the range9:    
*end if*
10:*end for*11:$I_{A}=I_{A}^{T}$

Finally, the pseudo-code of HAG-NET when generating $I_{E}$ and updating the parameters of each component is shown in Algorithm 2.
**Algorithm 2** HAG-NET generate adversarial embedding examples**Input:**Carrier image *I_co_*, target attack label *T_co_*, accompanying switch, secret message *M_in_*, pre-trained target classification network *C*, maximum training number *e*.**Output:**Encoder parameters ∅, decoder parameters γ and adversarial stego example $I_{E}$
1:$\emptyset^{0}$ ← rand, $\gamma^{0}$← rand, $\theta^{0}$←rand//parameter initialization2:*for  i ←1*,…, *e do*3:    
$M_{in}^{i}$
*← rand*
4:    *Generating*
$I_{A}$ *by Algorithm 1*5:    
$I_{E}^{i}$
* = *
$E_{\theta^{i-1}}\left(I_{co},M_{in}^{i}\right)$
6:    
$M_{out}^{i}$
* = *
$D_{\emptyset^{i-1}}\left(I_{E}^{i}\right)$
7:    
*If*  
*switch = True  then*
8:        
$\tilde{I}\in \left\{I_{A},I_{E}^{i}\right\}$
9:    
*Else*
10:        
$\tilde{I}\in \left\{I_{co},I_{E}^{i}\right\}$
11:    
*end if*
12:    
$L_{A}^{i}$
* = *
$L_{A}\left(A_{\gamma^{i-1}}\left(\tilde{I}\right)\right)$
13:    γ ← $\gamma^{i}$//Update the parameters γ to $\gamma^{i}$
14:    
$L_{E}^{i}$
* = *
$L_{E}\left(\tilde{I}\right)$
15:    
$L_{G}^{i}$
* = *
$L_{G}\left(A_{\gamma }\left(I_{E}^{i}\right)\right)$
16:    
$L_{D}^{i}$
* = *
$L_{D}\left(M_{in}^{i},M_{out}^{i}\right)$
17:    
$L_{C}^{i}$
* = *
$L_{C}\left(I_{E}^{i},T_{co}\right)$
18:    Backward $\lambda_{E}\times L_{E}^{i}+\lambda_{G}\times L_{G}^{i}+L_{D}^{i}+\lambda_{C}\times L_{C}^{i}$
19:    Update encoder’s parameters $\theta^{i}$ and decoder’s parameters $\emptyset^{i}$
20*end for*21:$\theta =\theta^{e},\;\emptyset =\emptyset^{e}$//Save the optimal parameters of encoder *E*, decoder *D*22:$I_{E}$* = *$E_{\theta }\left(I_{co},M_{in}\right)$//Output Adversarial Steganographic example 

## 4. Experiments and Results

### 4.1. Experimental Setting

The target classification network is most vulnerable to attacks under the white-box setting, wherein the adversary possesses complete knowledge of all its parameters. Therefore, we concentrate on evaluating HAG-NET’s attack capabilities against various target classification networks. For training on the MNIST dataset, we chose Model A [22] and Model B [6] as the target classification networks. Similarly, for the CIFAR-10 dataset, the target classification networks were ResNet32 and WRN34.

**Figure 4:**
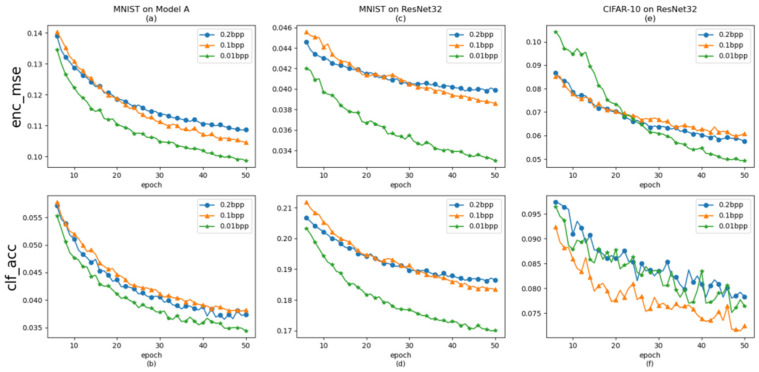
Pre-training of HAG-NET under different experimental settings, where (**a**,**b**) show the curves of *L_E_* loss and the classification accuracy of target classification network Model A in MNIST dataset, (**c**,**d**) show the curves of *L_E_* loss and the classification accuracy of the target classification network ResNet32 in the MNIST dataset, (**e**,**f**) show curves of *L_E_* loss and the classification accuracy of the target classification network ResNet32 in the CIFAR-10 dataset.

Figure 4a depict curves illustrating the Mean Squared Error (MSE), representing the $L_{2}$ norm distance between the Adversarial Steganographic Example $I_{E}$ and the original cover image $I_{CO}$, and Figure 4b depict the classification accuracy, indicating the success rates at which the target classification networks correctly classify images. These curves are generated under different settings after 50 epochs of pre-training. Specifically, they display the results for various embedding capacities (0.01BPP, 0.1BPP, and 0.2BPP) while training HAG-NET to attack Model A on MNIST (Figure 4a,b), ResNet32 on MNIST (Figure 4c,d), and ResNet32 on CIFAR-10 (Figure 4e,f). Lower MSE values signify better visual quality of the ASE, while decreased classification accuracy indicates improved adversarial attack efficacy of the ASE against the target classification network.

We primarily measure capacity in terms of bits per pixel (BPP), representing the number of secret message bits hidden per pixel of the Adversarial Steganographic Example (ASE), which is calculated as $L/\left(c\times H\times W\right)$. During the 50 epochs of pre-training, we observed a consistent downward trend in the Mean Squared Error (MSE) curves, as depicted in Figure 4a,c,e. This trend is also correlated with different embedding capacities. Specifically, the MSE associated with a 0.01 BPP embedding capacity consistently reached the lowest value after 50 epochs of pre-training, irrespective of the dataset, as shown in Figure 4a,c, or the target networks, as shown in Figure 4c,e. In other words, the ASEs generated with a 0.01 BPP setting are the most indistinguishable from the cover image. Although the curves of classification accuracy for different target classification networks and datasets also exhibit a similar downward trend, as depicted in Figure 4b,d,f, the curve of classification accuracy under a smaller embedding capacity setting is not always lower than that under a larger embedding capacity at the same epoch. For instance, the ASE generated with a 0.1 BPP achieved the lowest classification accuracy value in Figure 4f, but it also yielded the highest value in Figure 4b.

To achieve the optimal visual effect of Adversarial Steganographic Examples (ASEs), we will assess the attack effectiveness of HAG-NET under an embedding capacity setting of 0.01 BPP, which is independent of the attack capability. Specifically, for the CIFAR-10 dataset with images of size 32×32×3, the length *L* of $M_{IN}$ is set to 31 bits, whereas for MNIST, a grayscale image dataset with images of size 28×28×1, the length *L* of $M_{IN}$ is set to 8 bits.

### 4.2. Loss Funcationes Evaluation

**Figure 5:**
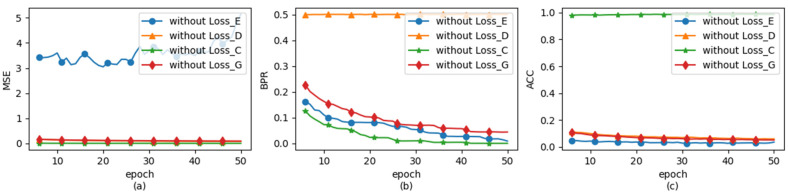
HAG-NET provides line graphs illustrating the variations in different types of data when each component loss function is individually removed. Among these, (**a**) illustrates the changes in Mean Squared Error (MSE) between ASE and the carrier image under various conditions; (**b**) displays the variations in Bit Error Rate (BER) of decoded information; and (**c**) demonstrates the changes in accuracy of target classification network in recognizing ASE.

To assess the influence of each loss function on the generation process of HGA-NET, we conducted experiments by individually removing $L_{E}$, $L_{D}$, $L_{G}$, and $L_{C}$ from the objective function of HGA-NET while keeping the rest unchanged. The resulting sample images and corresponding perturbations after 50 epochs of training are presented in Table 2 and Figure 5 for comparison.

From Table 2, we observe that upon removal of the $L_{E}$ constraint, the strength of adversarial embedding perturbations is no longer restricted, resulting in the degradation of the carrier image data. Similarly, as depicted in Figure 5a, it can be noted that the Mean Squared Error (MSE) between the adversarial steganographic embedding (ASE) generated by the $L_{E}$-unconstrained HAG-NET and the carrier image surpasses that of the removal of other loss functions. After removing the loss function $L_{G}$, the authenticity of ASE will be called into question. This is primarily because the absence of adversarial loss from discriminator *D* will lead to an increase in the divergence between the data of ASE and that of real images, resulting in an enlarged gap between their data distributions. For the loss functions $L_{D}$ and $L_{C}$, they ensure that ASE achieves both data hiding and adversarial robustness against the target classification network. When either one is removed, the other effect of ASE becomes more pronounced. As shown in Table 2 and Figure 5b,c, after removing $L_{C}$, ASE emphasizes data hiding more, resulting in better visual quality and smaller perturbation strength. However, the adversarial robustness of ASE towards the target classification network is completely lost. Conversely, removing $L_{E}$ shifts ASE’s focus towards adversarial robustness, leading to decreased visual quality, increased perturbation strength, and the inability of the decoder to extract any secret information from the perturbations. This also demonstrates why tuning the hyperparameters of the generator loss function can adjust the performance emphasis of Adversarial Steganographic Embedding (ASE).

### 4.3. White-Box Attack Evaluation

HAG-Net demonstrates remarkable performance in adversarial embedding, enhancing the efficiency of adversarial embedding, as illustrated in Table 3 below.

To enhance the adversarial effect of Adversarial Steganographic Examples (ASEs), we pre-train the encoder and decoder until the Bit Error Rate (BER) between $M_{IN}$ and $M_{OUT}$ is below $10^{-5}$. The Bit Error Rate (BER) is calculated by dividing the number of erroneously decoded bits by the length *L* of the secret message. This pre-trained network effectively embeds and extracts secret messages, employing an adversarial approach from the inception of training.

We randomly select 500 images from the MNIST and CIFAR-10 datasets for verification purposes, showcasing the target attack success rates of HAG-NET across different target classification networks. The simulations are conducted under the condition that the BER of the decoded message is less than $10^{-5}$, as detailed in Table 4.

On the CIFAR-10 dataset, HAG-NET achieves average success rates of 99.03% and 99.16% with ResNet32 and WRN34, respectively. Similarly, on the MNIST dataset, HAG-NET demonstrates outstanding performance, achieving success rates of 99.26% with Model A and 99.11% with Model B. Furthermore, HAG-NET achieves success rates above 98.40% with different target classification networks for any target classes, with the maximum success rate reaching 99.88%, above the minimum success rate of 1.44%. This result illustrates the robustness of HAG-NET’s adversarial attack effect across MNIST and CIFAR-10 datasets. Additionally, this robustness is corroborated by the average success rates when attacking both grayscale and RGB images, both exceeding 99%.

We primarily compare HAG-NET with recent generative adversarial attack methods, namely ADV-GAN and AI-GAN, as they emphasize adversarial attacks and share similarities with our approach. In Table 5, we blod the best average success rates achieved with different methods across various target classification networks and datasets. The results indicate that HAG-NET emerges as one of the top-performing generative adversarial attack methods under similar dataset and target classification network settings. Our method achieves the highest average success rates with Model A, Model B, and WRN 34. Compared with the worst-case scenario among the same target classification networks, HAG-NET demonstrates improvements of 1.36%, 0.81%, and 4.46%, respectively. Notably, compared to AI-GAN, which secured second place, HAG-NET achieves a 3.32% improvement under the same WRN34 setting. Conversely, ADV-GAN achieves the best average success rates with ResNet32, with HAG-NET trailing closely by only 0.27%. It is important to note that the adversarial attack effectiveness of ASEs also considers decoding error rates less than $10^{-5}$, imposing stricter constraints compared to other methods. Thus, we assert that HAG-NET exhibits superior adversarial attack effectiveness among them.

**Figure 6:**
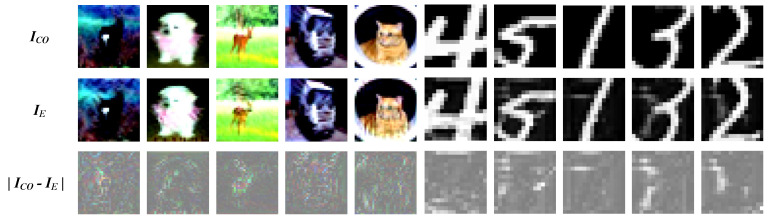
The ASE of 0–4 target class in CIFAR-10 and the ASE of 5–9 target class in MNIST.

The Figure 6 shows the ASE ($I_{E}$) of targeted classes 0–4 generated on the CIFAR-10 dataset and 5–9 targeted class generated on the MNIST dataset. In the Figure 6, $I_{CO}$ represents the cover images, and $\left|I_{CO}-I_{E}\right|$ indicates the perturbations between $I_{CO}$ and $I_{E}$.

**Figure 7:**
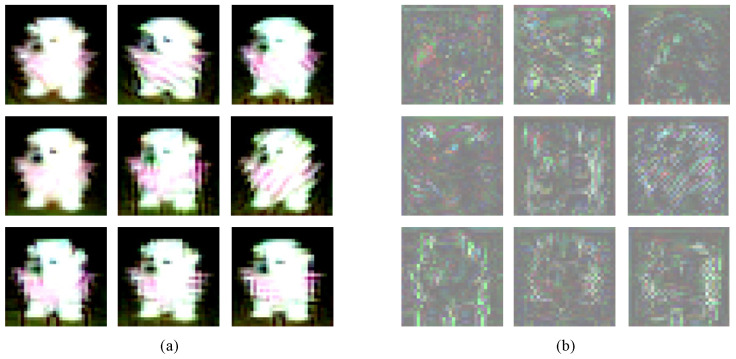
(**a**) shows the ASE of that a dog image has been attacked into remaining nine classes, from top to bottom and left to right they are plane, car, bird, cat, deer, frog, horse, ship, and truck. (**b**) shows the corresponding adversarial embedded disturbance at the same location.

Figure 7a illustrates a natural image of a dog being transformed into the $I_{E}$ of the remaining nine classes, arranged from top to bottom and left to right as follows: airplane, car, bird, cat, deer, frog, horse, boat, and truck. HAG-NET is capable of performing different target attacks on the same natural image, as depicted in Figure 7b. The corresponding perturbations are displayed at the same location. It can be observed that the disturbance generated by attacking a target class similar to the original class is relatively small compared to other classes. For instance, the original image of a dog being attacked and classified as a cat, shown in the first column of the second row in Figure 7b, produces an $I_{E}$ that is the most indistinguishable from $I_{CO}$.

### 4.4. Robustness Evaluation

In this subsection, we assess HAG-NET under the scenario where the target classification network is aware of potential attacks. Consequently, the target classification network will employ several commonly used defense methods proposed in [19] to counter adversarial attacks. These methods have been proven to significantly enhance the robustness of the target classification network. They include: (1) adversarial training with FGSM (Adv), (2) ensemble adversarial training (Ens), and (3) adversarial training with PGD.

The adversarial attack methods involved in this evaluation do not have access to the specific parameters of the target classification networks or the defense mechanisms employed by them. Additionally, during the training process of these attack methods, the target classification networks are replaced by vanilla models. The experimental results comparing HAG-NET, PGD, ADV-GAN, and AI-GAN under various defense methods for the target classification networks are presented in Table 6 below.

Through calculation, the average success rates of PGD, ADV-GAN, AI-GAN, HAG-NET (A), and HAG-NET (B) are 11.05%, 10.09%, 12.92%, 11.29%, and 13.06%, respectively. As depicted in Table 4, it is evident that the success rates of HAG-NET (B) across different defense methods and datasets either match or outperform other methods. Notably, the success rates of HAG-NET (A) rank in the top two only for ResNet32 with the Iter-Adv defense method and Model B with the Adv defense method, which are generally lower than other adversarial attack methods, except ADV-GAN. This observation is further supported by their average success rates. However, compared to HAG-NET (A), the success rates achieved by HAG-NET (B) trained with PGD attacker demonstrate a significant enhancement, with an average improvement of 2.91% on Model A and Model B. Nevertheless, it is regrettable that the improvement of HAG-NET (B) is only 0.41% on CIFAR-10. We posit that HAG-NET (B) may inherit a certain degree of robustness from the AE generated by the PGD method on the MNIST dataset, given that the performance of the PGD method on the CIFAR-10 dataset is also suboptimal, which explains the small improvement on CIFAR-10.

### 4.5. Data Hiding and Image Information Entropy

We compare the Bit Error Rate (BER) of HAG-NET with classical watermarking algorithms such as HUGO, WOW, and HiDDeN, as well as with the latest adversarial embedding method ADV-EMB, which shares similar functionalities with ours, across various embedding capacities. The Bit Error Rate (BER) of decoded messages serves as the experimental index value, as illustrated in Table 7 below.

Unfortunately, HAG-NET still exhibits the characteristic decoding errors that are shared with the latest generative watermarking methods, HiDDeN and ADV-EMB. At an embedding capacity setting of 0.101 bits per pixel (BPP), the Bit Error Rate (BER) of the decoded message for both our method and HiDDeN remains below $10^{-5}$. However, when the embedding capacity setting increases to 0.203 BPP, the BER of HAG-NET rises to $10^{-3}$. However, under the setting of 0.203 bits per pixel (BPP), HAG-NET performs consistently with ADV-EMB in terms of performance. It is noteworthy that, compared to the perturbations generated by ADV-EMB, which are only adversarial against binary steganalysis, HAG-NET achieves adversarial robustness against larger classification networks.

To determine whether Adversarial Steganographic Examples (ASEs) contain more information than other watermarking methods based on image information entropy, we selected some samples generated by our method and others with similar disturbance intensity and the same embedding capacity setting for calculating image information entropy. This process aims to ensure that Deep Neural Networks (DNNs) perceive these samples generated by different methods similarly and that they possess the same level of information as perceived by human eyes. The results are presented in Table 6.

From Table 8, it is evident that the samples generated by all watermarking methods exhibit an increase in image entropy compared to that of the carrier image, indicating an augmentation in the amount of information contained within the images. Across all embedding capacities, adversarial embedding methods consistently demonstrate higher information content than HiDDeN, implying that adversarial information is effectively embedded into the carrier image in an imperceptible manner. While at 0.101 BPP, the MSE of ASE produced by HAG-NET exceeds that of ADV-EMB, at 0.010 BPP, HAG-NET still maintains the highest image entropy despite having a lower MSE compared to ADV-EMB. We attribute this observation to the adversarial perturbations generated by our method containing more information when confronted with larger, more complex networks compared to the binary classification network detection countered by ADV-EMB.

## 5. Conclusions

In this paper, we introduce a novel generative adversarial watermarking model, HAG-NET, which is jointly trained by an encoder, decoder, and an attacker. The Adversarial Steganographic Examples (ASEs) generated by the trained HAG-NET can embed secret messages and achieve protection against detection for the carrier data. Compared with recent generative watermarking or attack methods, our approach demonstrates comparable or superior performance. We verified that under the same disturbance intensity, the perturbation of ASEs consistently contains more information than perturbations generated by other methods across various settings such as embedding capacity and dataset.

## Figures and Tables

**Table 1:** The network frameworks of ResNet32 and WRN34.

ResNet 32	WRN 34
conv2d layer(kernel = 3, stride = 1, depth = 16)	conv2d layer(kernel = 3, stride = 1, depth = 16)
basic block layer1 = basic block(16) × 5	basic block layer1 = basic block(16, 160) × 5
(basic block(16,16):	(basic block:
conv2d layer(kernel = 3, stride = 1, depth = 16)	batch norm layer(eps = 0.00001, depth = 16)
batch norm layer(eps = 0.00001, depth = 16)	conv2d layer(kernel = 3, stride = 1, depth = 160)
conv2d layer(kernel = 3, stride = 1, depth = 16)	batch norm layer(eps = 0.00001, depth = 160)
batch norm layer(eps = 0.00001, depth = 16)	conv2d layer(kernel = 3, stride = 1, depth = 160)
shortcut)	shortcut:)
basic block layer2 = basic block(32) × 5	basic block layer2 = basic block(160, 320) × 5
basic block layer3 = basic block(64) × 5	basic block layer3 = basic block(320, 640) × 5
average pooling layer(kernel- = 6, stride = 1)	average pooling layer(kernel- = 6, stride = 1)
flatten layer	flatten layer
softmax classifier	softmax classifier

**Table 2:** A schematic diagram depicting the partial loss functions during the training process of HGA-NET is absent.

Train Without	*L_E_*	*L_D_*	*L_G_*	*L_C_*
*I_E_*	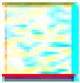	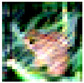	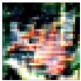	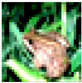
|*I_CO_* – *I_E_*|	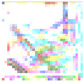	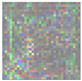	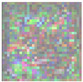	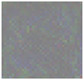

**Table 3:** Runtimes of HAG-NET, FGSM, C&W, PGD, and HUGO.

	FGSM	C&W	PGD	HUGO	HAG-NET
Runtime	0.06 s	>3 h	0.7 s	0.08 s	<0.01 s

**Table 4:** The attack success rate of target attacks by HAG-NET on each target classification networks in MNIST and CIFAR-10 datasets.

	MNIST		CIFAR-10	
Target Class	Model A	Model B	ResNet32	WRN34
Class 0	98.58%	99.69%	98.88%	98.98%
Class 1	99.04%	98.32%	99.68%	99.38%
Class 2	99.25%	98.80%	99.16%	99.36%
Class 3	99.88%	98.65%	99.50%	99.16%
Class 4	98.79%	99.00%	98.40%	99.30%
Class 5	99.40%	99.72%	99.48%	99.10%
Class 6	99.21%	99.42%	98.88%	99.32%
Class 7	99.65%	99.33%	98.91%	99.35%
Class 8	98.98%	99.17%	98.76%	98.83%
Class 9	99.83%	99.04%	98.65%	98.85%
Average	99.26%	99.11%	99.03%	99.16%

**Table 5:** The average attack success rate of ADV-GAN, AI-GAN, and HAG-NET to target attack Model A, Model B, ResNet32, and WRN34 on MNIST and CIFAR-10 datasets.

	MNIST		CIFAR-10	
Methods	Model A	Model B	ResNet32	WRN34
ADV-GAN	97.90%	98.30%	**99.30%**	94.70%
AI-GAN	99.14%	98.50%	95.39%	95.84%
HAG-NET	**99.26%**	**99.11%**	99.03%	**99.16%**

**Table 6:** The success rates of different adversarial attack methods against a target classifier with defense mechanisms.

	MNIST						CIFAR-10					
	Model A			Model B			ResNet32			WRN34		
Methods	Adv.	Ens.	Iter.Adv	Adv.	Ens.	Iter.Adv	Adv.	Ens.	Iter.Adv	Adv.	Ens.	Iter.Adv
PGD	20.59	11.45	**11.08**	10.67	10.34	9.90	9.22	10.06	**11.41**	8.09	9.92	9.87
Adv-GAN	8.00	6.30	5.60	18.70	**13.50**	12.60	10.19	8.96	9.30	9.86	9.07	8.99
AI-GAN	**23.85**	**12.17**	10.90	**20.94**	10.73	**13.12**	9.85	**12.48**	9.57	**10.17**	**11.32**	9.91
HAG-NET(A)	15.37	10.65	7.16	15.49	10.02	13.03	**10.78**	12.02	10.99	9.64	10.00	**10.33**
HAG-NET(B)	**19.60%**	**11.88**	**11.56**	**19.91**	**11.30**	**15.23**	**10.30**	**11.45**	**12.10**	**10.05**	**12.17**	**11.16**

HAG-NET(A) is HAG-NET without attacker and HAG-NET(B) is HAG-NET with attacker. All data in this table are presented in % unit, and the top two results of each experiment are shown in bold.

**Table 7:** BRE differences between HAG-NET and other data hiding methods under different capacity settings.

Method	BPP	BER	BPP	BER	BPP	BER
**HUGO**	0.010	0	0.100	0	0.200	0
**WOW**	0.010	0	0.100	0	0.200	0
**HiDDeN**	0.011	<10^−5^	0.101	<10^−5^	0.203	<10^−5^
**ADV-EMB**	0.011	<10^−5^	0.101	<10^−5^	0.203	<10^−3^
**HAG-NET**	0.011	<10^−5^	0.101	<10^−5^	0.203	<10^−3^

**Table 8:** The difference of MSE and information entropy value between HAG-NET and others.

	0.010 BPP			0.101 BPP		
	MSE	IMAGE	ENTROPY	MSE	IMAGE	ENTROPY
I_CO_	0	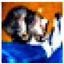	≈6.793	0	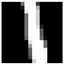	≈1.847
HiDDeN	0.0042	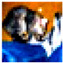	≈7.037	0.8120	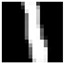	≈5.078
ADV-EMB	0.0045	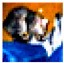	≈7.046	0.8319	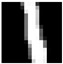	≈5.158
HAG-NET	0.0040	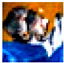	≈7.056	0.8550	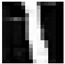	≈5.319

## Data Availability

No new data were created or analyzed in this study. Data sharing is not applicable to this article.
